# Healthful Drinks

**Published:** 1874-06

**Authors:** 


### HEALTHFUL DRINKS.

ONE of the crowning blessings of the United States, in comparison with Continental Europe, is the delicious and wholesome character of the drinking water. Whether it is because so many people have been buried in the Old World, and the filth of six thousand years scattered over the surface, has corrupted the waters of all the springs and wells, need not be discussed here; certain it is that European water, not excepting any part of Great Britain, except in spots, is execrable, while water in our country is simply delicious. They may require beer and wine over there; we do not need them at all; and as the step between beer and brandy and wine and whiskey is short, certain and inevitable, and inasmuch as there are certain conditions of the system and seasons which may make it expedient that something more than pure water be drank, especially in the harvest field, and under kindred circumstances, when nutriment and strength is demanded, besides the mere slaking of the thirst; it is well to know some of the mixtures which supply these requirements in a safe, healthful and agreeable manner.

Fresh buttermilk answers to all these needs, and it is much to be regretted that there are many farmers, even in intelligent New England, who consider it unfit for human food, and give it to the pigs. Our German friends know better, for they learned in the Fatherland the virtues of sour milk—the habit of many there being to curdle sweet milk before putting it on the table. Many Germans in Cincinnati use buttermilk almost exclusively.

A pint of molasses in two gallons or a pail of water, at the temperature of the surrounding atmosphere, even in midsummer, is a safer, more cooling, more wholesome drink than the purest spring water. Two teaspoonfuls of raw, fresh oatmeal, stirred in a tumbler of cool water, is a delicious and nutritious drink, or a quart of this meal in a pail of spring water.

